# Surgical treatment of intraperitoneal metastases from lung cancer: two case reports and a review of the literature

**DOI:** 10.1186/s13256-019-2178-5

**Published:** 2019-08-21

**Authors:** Simone Sibio, Giuseppe Sigismondo Sica, Sara Di Carlo, Maurizio Cardi, Alessandra Di Giorgio, Bianca Maria Sollazzo, Paolo Sammartino

**Affiliations:** 1 Department of Surgery “Pietro Valdoni”, “Sapienza” University of Rome, Via Lancisi 2, 00155 Rome, Italy; 2 Department of Surgery, Tor Vergata University of Rome, Viale Oxford 81, 00133 Rome, Italy

**Keywords:** Lung cancer, Peritoneal metastases, Cytoreductive surgery

## Abstract

**Background:**

Peritoneal metastases are often reported in several abdominal tumors. Peritoneal diffusion from extra-abdominal tumors is thought to be rare. Lung cancer is one of the most common cancers in the world with early metastases and it is associated with poor prognosis in advanced stages. Peritoneal metastases from lung cancer are uncommon and the real mechanism of its diffusion to the peritoneum is unknown. However, its clinical behavior is similar to any other peritoneal metastasis from abdominal tumors.

**Case presentation:**

We present two Caucasian patients (a 44-year-old man and a 59-year-old man) with bowel obstruction from peritoneal metastases from non-small cell lung cancer who successfully underwent emergency cytoreductive surgery and had a good prognosis and survival.

**Conclusions:**

In our patients with isolated peritoneal metastases from lung cancer, cytoreduction showed good prognosis with acceptable morbidity. This treatment option might be considered in highly selected cases to improve survival. Strict follow-up is mandatory to allow early diagnosis of peritoneal diffusion.

## Background

The occurrence of peritoneal metastases represents the clinical final stage of several tumors: most commonly ovarian, colorectal, gastric cancers, and, less frequently, appendix cancer. In recent years, the inclusion of cytoreductive surgery, alone or associated with locoregional chemotherapy, in the treatment strategy of some of these conditions has increased (peritoneal metastases from ovarian cancer or primary peritoneal malignancies), whereas its application remains mainly investigational in other conditions (gastric, colorectal, endometrial, and breast cancers) [1, 2].

Lung cancer is one of the most common cancers worldwide. It is the leading cause of death from cancer; diagnosis is often made at an advanced stage when metastases have already spread to the other lung, to the liver, to the brain, to the bone, and to adrenal glands [3].

Few reports are available in the literature about patients with peritoneal metastases from lung cancer [4–6]. Despite any treatment, overall survival for these patients is very poor, with reported rates of 9 months in a recent series [5].

We describe two cases of diffused peritoneal metastases from lung cancer who underwent emergency cytoreductive surgery for bowel occlusion and had an unexpectedly good prognosis and long-term survival.

## Cases presentation

### Patient 1

**Fig. 1:**
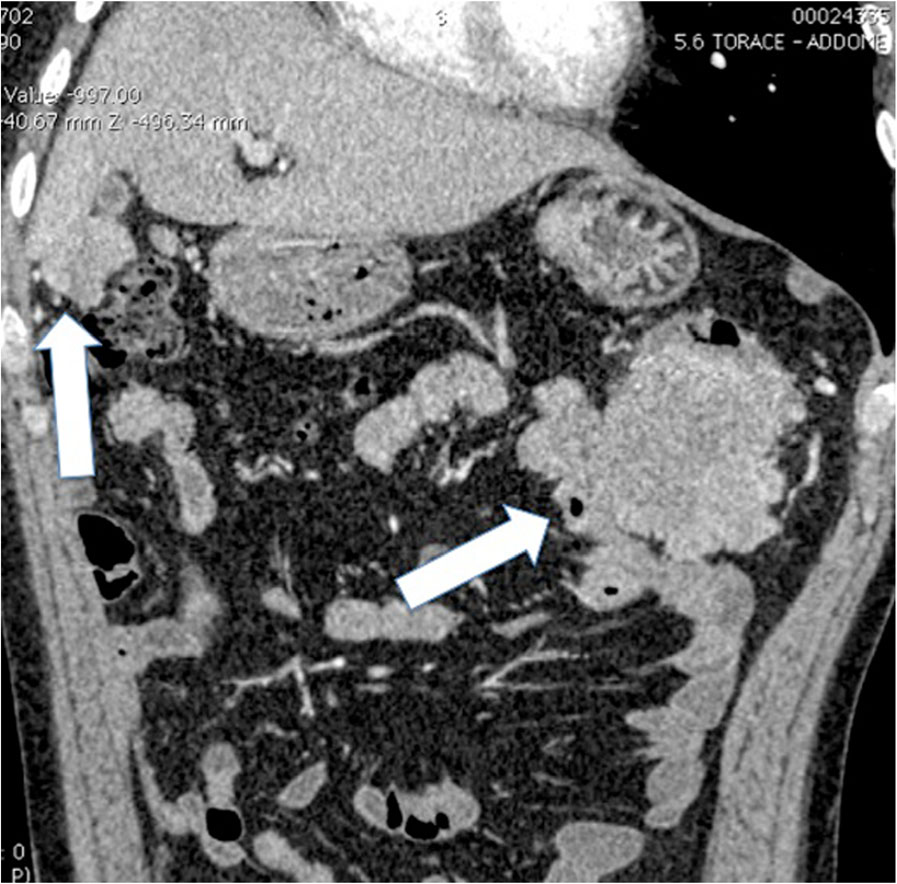
Coronal two-dimensional image showing huge implants (*arrows*) of peritoneal metastases located near colonic splenic flexure providing a compression of the lumen and in Morison pouch between right kidney and right liver

**Fig. 2:**
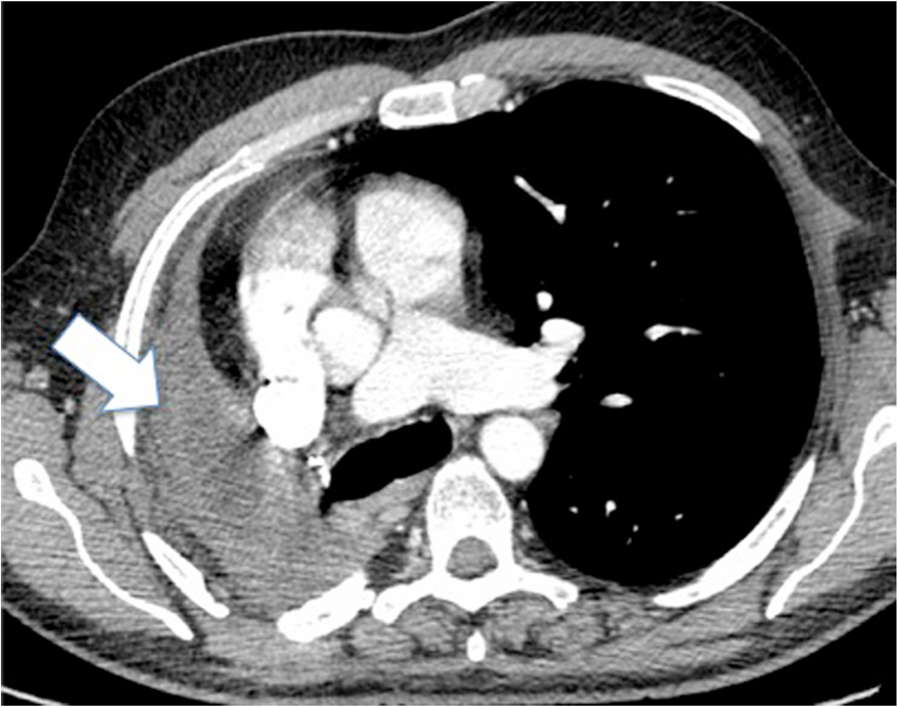
Axial two-dimensional image obtained after intravenous administration of iodinated contrast agent, showing outcomes of right pneumonectomy (*arrow*)

In March 2013, a 44-year-old Caucasian man, a non-smoker of tobacco, was referred to our department as an emergency with 3 days’ history of bowel obstruction. His family history was negative for cancer. His past medical history included a right pneumonectomy in 2009 for a T2, N1, M0 G3, stage IIB lung adenocarcinoma. Immunohistochemistry was positive for cytokeratin 7 and negative for thyroid transcription factor 1 (TTF-1), caudal type homeobox transcription factor 2 (CDX2), cytokeratin 20, protein S100, thyroglobulin, the anti-melanosome clone, human melanoma black 45 (HMB-45), and the anti-melanoma, melanoma antigen recognized by T cells 1 (MART-1). After surgery, he underwent four cycles of adjuvant chemotherapy with cisplatin (100 mg/m^2^) and paclitaxel (175 mg/m^2^); he then received two cycles of gemcitabine (1000 mg/m^2^) after the fourth cycle of cisplatin and paclitaxel for a grade 4 toxicity to paclitaxel. On admission in our department, he was off any treatment, and 1 month previously he had negative magnetic resonance imaging (MRI) for brain metastases. His vital signs were normal. Performance status assessed by the Eastern Cooperative Oncologic Group (ECOG) [7] was 1. He had been vomiting and his bowel had been obstructed for 24 hours. A clinical examination revealed a tender distended abdomen. Blood tests were normal except for neutrophilic leukocytosis: white blood cells (WBC) 14,000/mm^3^. Neuron-specific enolase and cytokeratin-19 fragment (CYFRA 21-1) levels were normal (respectively 121 ng/ml and < 3 ng/ml). A total body contrast-enhanced computed tomography (CT) scan showed a distended large bowel with air-fluid levels and multiple neoplastic implants involving the right colon, greater omentum, spleen, and the sigmoid colon, ranging from 0.5 to 10 cm. (Fig. 1). No other pathological findings were disclosed and a chest examination was negative except for the outcomes of the previous thoracic surgery (Fig. 2).

**Fig. 3:**
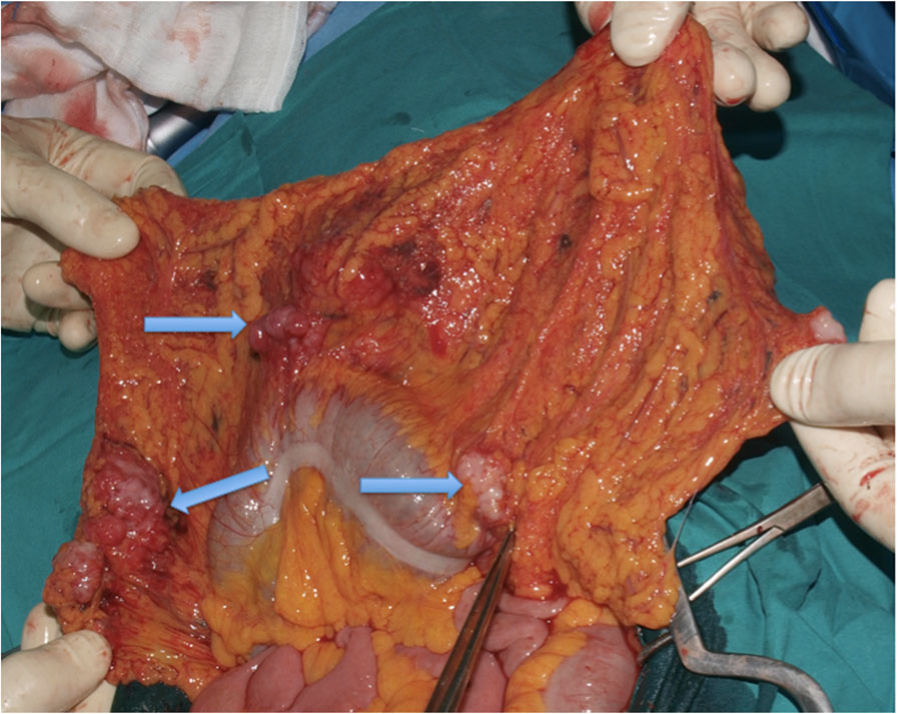
Intraoperative picture showing gross neoplastic implants on greater omentum, transverse colon, and left colon (*arrows*)

**Fig. 4:**
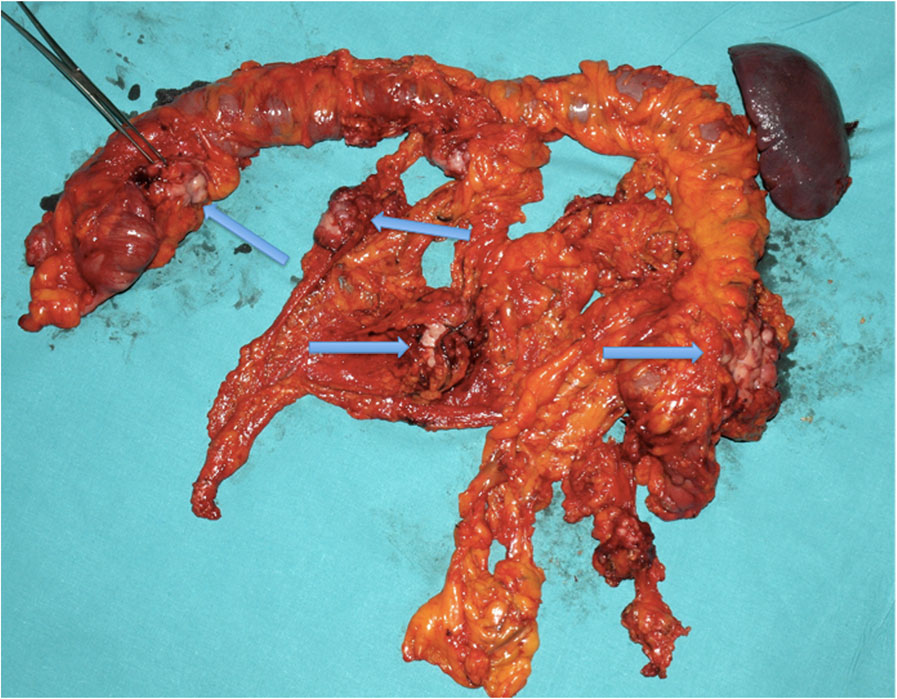
Final surgical specimen showing gross neoplastic implants involving right side of colon, transverse colon, left colon, greater omentum, and splenic flexure (*arrows*)

Considering his young age, the absence of lung recurrence and of any other distant metastasis, palliative surgery was considered in order to treat bowel obstruction. At laparotomy there was no ascites, and three gross neoplastic implants were found in the greater omentum, right colonic flexure, transverse colon, and left colon. Extensive cytoreductive surgery was performed and surgical procedures included subtotal colectomy with ileosigmoid anastomosis, splenectomy, and greater omentectomy (Figs. 3 and 4). At the end of the procedure no residual macroscopic disease was left, reaching a completeness of cytoreduction score (CCS) of 0.

Pathology showed complete infiltration of the colonic wall and spleen by adenocarcinoma nodules with lymph node metastases in the mesocolon.

Immunohistochemistry evaluation showed the same staining as the previous lung adenocarcinoma, confirming the lung origin of the peritoneal metastases.

His postoperative course was complicated by fever (38.5 °C) and dyspnea on postoperative day 9. A chest X-ray showed a “ground glass” picture of left lung and bloodstream and expectorate cultures were positive for *Candida albicans* species. Intravenously administered anidulafungin treatment was started with rapid improvement of our patient’s general condition and gradual resolution of sepsis. He was discharged on postoperative day 20 and subsequently he underwent six cycles of adjuvant chemotherapy with cisplatin (100 mg/m^2^). Considering the previous toxicity to paclitaxel no other drug was used. A follow-up protocol included clinical evaluation (1 month after surgery, then every 3 months), blood tests with tumor marker levels every 3 months, and total body CT scan 1 month after surgery, then every 3 months for the first year and every 6 months for the next 2 years. Yearly, a brain MRI was scheduled. He was alive and disease free 3 years after surgery. In September 2016 he was lost to follow-up.

### Patient 2

In September 2011, a 59-year-old Caucasian man, a heavy tobacco smoker, presented to our department as an emergency with abdominal pain and vomiting. His past medical history included a left upper lobectomy for a T1N1M0, G3 stage IIB lung adenocarcinoma (3 years before) followed by six cycles of systemic chemotherapy (carboplatin + paclitaxel 175 mg/m^2^). His family history was positive for cancer (a 39-year-old brother died of colon cancer). Two days before admission, abdominal distension and bowel obstruction occurred and progressively worsened. His WBC count was 18,000/mm^3^. A total body CT scan showed a large mass involving the distal ileal loops with obstruction and distension of the proximal bowel, with a small amount of ascites in his pelvis.

He underwent explorative laparoscopy that confirmed the CT scan findings, showing no other peritoneal seeding. Ascites was taken for cytological examination, and a laparoscopic ileocolic resection with ileotransverse anastomosis was performed, reaching a CCS of 0. His postoperative course was uneventful and he was discharged on postoperative day 4. Pathology confirmed the diagnosis of metastasis from adenocarcinoma; ascites was found to be negative for neoplastic cells. At immunohistochemistry, cancer cells were positive for cytokeratin 7 and TTF-1 confirming the origin of peritoneal metastases from the lung cancer. He was followed by medical oncologists and, due to his poor general condition, he underwent a second-line adjuvant chemotherapy with gemcitabine only (1000 mg/m^2^). He was disease free for 2 years. Subsequently, brain metastases occurred and in February 2014 he died (Table 1).

**Table 1:** Key features of the patients

	Patient 1	Patient 2
Age (years)	44	59
Sex	M	M
Primary lung cancer	T2 N1 M0 NSCLC	T1 N1 M0 NSCLC
Previous thoracic surgery	Right pneumonectomy	Left upper lobectomy
Previous adjuvant chemotherapy	Four cycles intravenously administered 100 mg/m^2^ cisplatin + 175 mg/m^2^ Taxol (paclitaxel) and Two cycles intravenously administered 1000 mg/m^2^ gemcitabine	Six cycles intravenously administered carboplatin + 175 mg/m^2^ paclitaxel
Clinical presentation	Bowel obstruction	Bowel obstruction
Surgical procedure	Subtotal colectomy, splenectomy, omentectomy	Laparoscopic ileocolic resection
CCS	0 (no residual disease)	0 (no residual disease)
Adjuvant chemo	Six cycles intravenously administered 100 mg/m^2^ cisplatin	Intravenously administered 1000 mg/m^2^ gemcitabine
Follow-up	Alive disease free	Died brain metastases
Survival (months)	36	25

*CCS*completeness of cytoreduction score, *M* male, *NSCLC* non-small cell lung carcinoma

## Discussion

Overall median survival in metastatic lung cancer is very poor. In recent studies it ranged between 3 and 12 months, depending on type of treatment [8]. Peritoneal metastases from extra-abdominal cancers are rare and have a poor prognosis. In a recent large population study by Flanagan *et al.*, the overall incidence rate of peritoneal metastases from extra-abdominal cancers was 9%, mostly originating from breast cancer (40.8%), followed by lung cancer (25.6%), and melanoma (9.5%) [5]. Satoh *et al*. reported a rate of 1.2% of peritoneal metastases complicating the clinical course of advanced lung cancer [9], while in other autopsy series this rate was found to be 12% [10]. Therefore, it could be argued that a number of cases of peritoneal metastases remains undiagnosed or unreported in patients with lung cancer. A few studies reported isolated bowel metastases from non-small cell lung carcinoma (NSCLC) with very poor prognosis [4, 9, 10] and some others described the peritoneal diffusion of pleural mesothelioma [11, 12].

In gastrointestinal tumors, peritoneal metastases generally arise either from direct invasion of the bowel wall or from cancer cells spilled by surgical manipulation [13]. These mechanisms are not applicable to lung cancer. In stage IV lung cancer, pleural metastases at diagnosis are significantly associated with subsequent peritoneal spread, whereas no association between oncogene status and peritoneal disease has been reported [6]. Pleural serosa infiltration might eventually explain the peritoneal seeding, but this mechanism could not be considered in our cases since our patients had no pleural disease.

According to the two most recent systematic reviews, bowel obstruction is the most frequent clinical presentation although bleeding and perforation are also reported [14, 15]. CT and positron emission tomography (PET) scans are useful tools for diagnosis in the late stages, while CT scan sensitivity is low at the early stage of peritoneal diffusion. In most patients, the time interval between primary lung cancer and peritoneal metastases ranges from 2 months to 4 years. The most frequent histology is NSCLC, with large cell cancer and adenocarcinoma being the most common subtypes. The prognosis for patients with peritoneal metastases from lung cancer is very poor regardless of the treatment, with a median survival rate of 2 to 4 months. In a recent review, Balla *et al.* found two cases of peritoneal metastases out of a sample of 91 patients with lung cancer with gastrointestinal metastases: the disparity with autopsy series suggests that most cases are asymptomatic or unreported [16, 17]. Emergency surgery for bowel obstruction and extra-abdominal metastases, found in up to 60% of patients [11, 12], could be the main reasons for the poor prognosis. However, the patients reported in this study had an unexpected good outcome despite their emergency presentation and the presence of diffused peritoneal involvement in one of them. They reached, respectively, 3-year and 2-year disease-free survival and one of them is currently alive. The absence of extra-abdominal metastases might perhaps explain the unexpected long survival. However, our results suggest that a combined approach (surgery and chemotherapy) could be advocated in selected patients with peritoneal metastases from lung cancer with some survival advantages compared to standard treatment. Cytoreductive surgery combined with hyperthermic intraperitoneal chemotherapy (HIPEC) in unconventional indications is occasionally reported in experienced tertiary centers [2], most frequently from rare ovarian cancers, sarcoma, or neuroendocrine tumors, and, more rarely, from gastrointestinal stromal tumor (GIST), hepatocellular and cholangiocarcinoma and desmoplastic small round cell tumors. The gap existing between the small reported series data and the incidence rates in autopsy series could suggest that in most cases peritoneal metastases from lung cancer remain clinically silent [16]. Our results on these two patients might help in stimulating more awareness of this condition and in suggesting a strict follow-up: in fact, early diagnosis of peritoneal diffusion, which is probably often underrated, could allow a radical cytoreductive surgery providing some advantages to survival.

Considering the behavior of lung cancer, in particular, its tendency to early metastases because of its continuous dynamic state and large blood and lymphatic supply that spreads a large amount of neoplastic cells directly in the bloodstream [18], the association of a strict follow-up and focused imaging techniques could help to identify selected cases to be treated, avoiding emergency “salvage” treatments that are difficult to perform even in dedicated centers.

High postoperative morbidity should be carefully considered when an extensive surgical treatment is planned on a patient with an advanced oncological stage. In our small series of two patients no major complications were observed, and maximal cytoreduction and HIPEC should not be discarded a priori as a treatment option. Further studies on this specific subset of patients could better clarify indications and limits.

## Conclusions

In our patients with isolated peritoneal metastases from lung cancer, cytoreduction showed good prognosis with acceptable morbidity. Although resection is the standard of care in bowel obstructions, this treatment option might be considered even in an elective stetting in highly selected cases to improve survival. Strict clinical and imaging diagnostic follow-up should be performed in these patients, and a peritoneal diffusion of the tumor should be anticipated and investigated.

## Data Availability

The datasets used and/or analyzed during the current study are available from the corresponding author on reasonable request.

## Abbreviations

CCS: Completeness of cytoreduction score
CDX2: Caudal type homeobox transcription factor 2
CT: Computed tomography
CYFRA 21-1: Cytokeratin-19 fragment
ECOG: Eastern Cooperative Oncologic Group
GIST: Gastrointestinal stromal tumor
HIPEC: Hyperthermic intraperitoneal chemotherapy
HMB-45: Human melanoma black 45
MART-1: Melanoma antigen recognized by T cells 1
MRI: Magnetic resonance imaging
NSCLC: Non-small cell lung carcinoma
PET: Positron emission tomography
TTF-1: Thyroid transcription factor 1
WBC: White blood cells
